# Targeted BRD4 protein degradation by dBET1 ameliorates acute ischemic brain injury and improves functional outcomes associated with reduced neuroinflammation and oxidative stress and preservation of blood–brain barrier integrity

**DOI:** 10.1186/s12974-022-02533-8

**Published:** 2022-06-27

**Authors:** Lei Liu, Changjun Yang, Bianca P. Lavayen, Ryland J. Tishko, Jonathan Larochelle, Eduardo Candelario-Jalil

**Affiliations:** Department of Neuroscience, McKnight Brain Institute, University of Florida, 1149 SW Newell Drive, Gainesville, FL 32610 USA

**Keywords:** Blood–brain barrier, BRD4, Neuroinflammation, Neurological deficits, Neuroprotection, Neutrophil infiltration, Oxidative stress, Stroke

## Abstract

Bromodomain-containing protein 4 (BRD4), a member of the bromodomain and extra-terminal domain (BET) protein family, plays a crucial role in regulating inflammation and oxidative stress that are tightly related to stroke development and progression. Consequently, BRD4 blockade has attracted increasing interest for associated neurological diseases, including stroke. dBET1 is a novel and effective BRD4 degrader through the proteolysis-targeting chimera (PROTAC) strategy. We hypothesized that dBET1 protects against brain damage and neurological deficits in a transient focal ischemic stroke mouse model by reducing inflammation and oxidative stress and preserving the blood–brain barrier (BBB) integrity. Post-ischemic dBET1 treatment starting 4 h after stroke onset significantly ameliorated severe neurological deficits and reduced infarct volume 48 h after stroke. dBET1 markedly reduced inflammation and oxidative stress after stroke, indicated by multiple pro-inflammatory cytokines and chemokines including IL-1β, IL-6, TNF-α, CCL2, CXCL1 and CXCL10, and oxidative damage markers 4-hydroxynonenal (4-HNE) and gp91^phox^ and antioxidative proteins SOD2 and GPx1. Meanwhile, stroke-induced BBB disruption, increased MMP-9 levels, neutrophil infiltration, and increased ICAM-1 were significantly attenuated by dBET1 treatment. Post-ischemic dBET1 administration also attenuated ischemia-induced reactive gliosis in microglia and astrocytes. Overall, these findings demonstrate that BRD4 degradation by dBET1 improves acute stroke outcomes, which is associated with reduced neuroinflammation and oxidative stress and preservation of BBB integrity. This study identifies a novel role of BET proteins in the mechanisms resulting in ischemic brain damage, which can be leveraged to develop novel therapies.

**Supplementary Information:**

The online version contains supplementary material available at 10.1186/s12974-022-02533-8.

## Introduction

Ischemic stroke insults trigger a complex pathophysiological cascade of events, including inflammation, oxidative stress, and blood–brain barrier (BBB) disruption that are tightly related to the extent of stroke brain damage and recovery [1]. Following stroke, the excessive production of inflammatory and oxidative mediators causes BBB disruption, peripheral immune cell infiltration, upregulation of cellular adhesion molecules, and the activation of glial cells, ultimately leading to exacerbated brain injury [2]. In recent years, increasing focus has been placed on developing therapeutic strategies to modulate inflammatory and oxidative status after stroke [3, 4].

Bromodomain-containing protein 4 (BRD4) is a member of the bromodomain and extra-terminal domain (BET) protein family that is widely distributed throughout the body [5, 6]. Recent structural and chemical analyses of BRD4 and its inhibition studies indicate a critical role of BRD4 in coordinating inflammatory and oxidative processes in response to different insults, including ischemia [5, 7–10]. These works include the identification of JQ1, a first-generation, potent, and selective BRD4 inhibitor [6]. A landmark study revealed that BET inhibition with I-BET potently suppressed the production of pro-inflammatory mediators in activated macrophages in vivo and protected against lipopolysaccharide (LPS)-induced lethal shock [11]. Several lines of evidence using in vitro oxidative stress models have revealed the protective role of BET inhibition against oxidative damage through upregulating the Nrf2 signaling pathway and its target antioxidant genes, suggesting that BET proteins inhibits Nrf2 signaling [12–15]. Following primary ischemic injury, secondary inflammatory and oxidative damage gradually occurs as a consequence of destructive cellular and molecular events, which further exacerbates BBB breakdown through various structural and functional components of the BBB [2, 16, 17]. Therefore, BRD4 blockade has attracted increasing interest in translational and clinical fields. However, the understanding of BRD4’s cellular functions and its relevance in diseases such as stroke remains limited, delaying advancements in translating therapeutic strategies targeting BRD4 to the clinic.

**Fig. 1:**
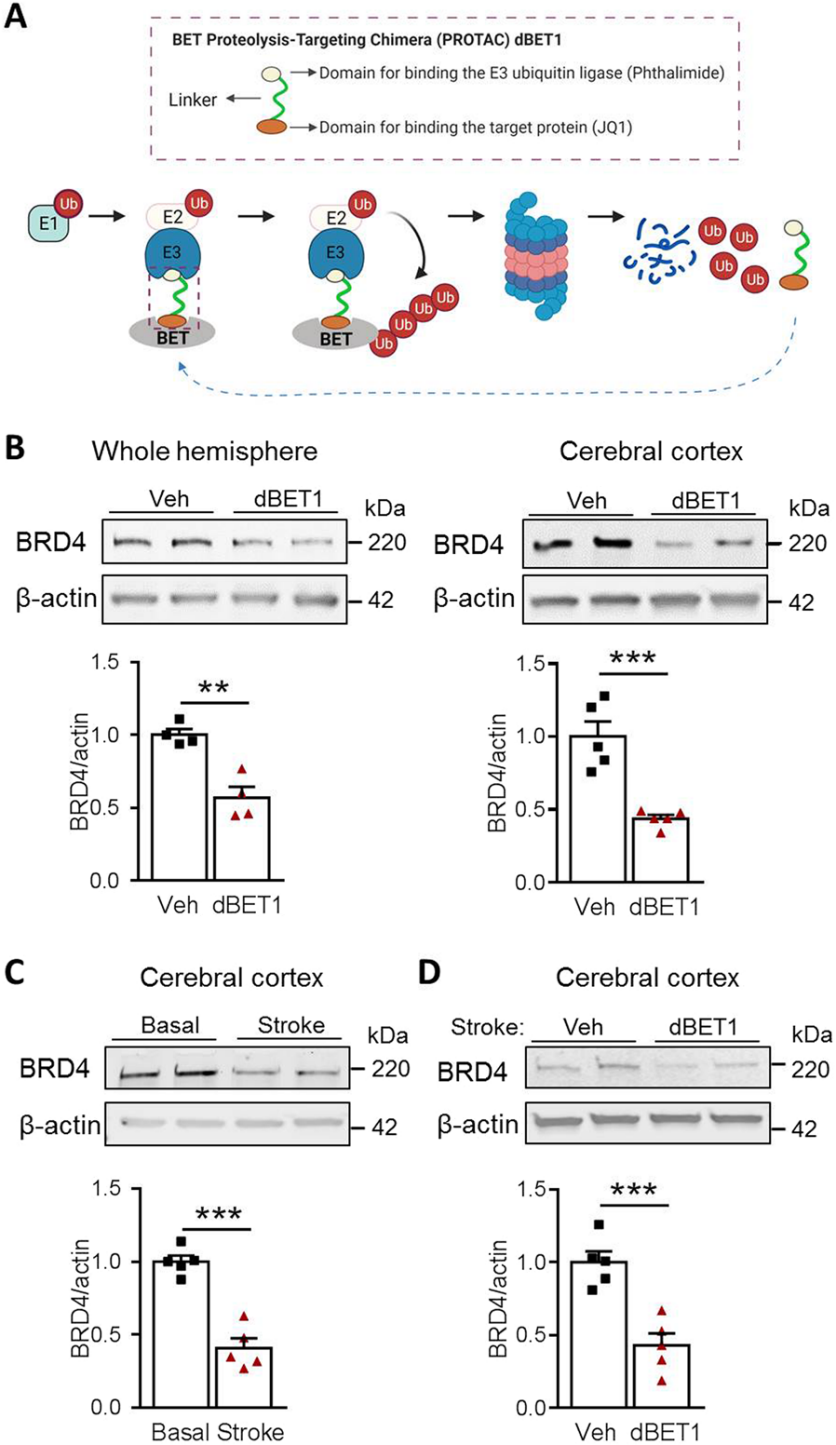
BRD4 protein degradation in the mouse brain by dBET1. **A** Schematic illustration of BRD4 PROTAC dBET1 mechanism-induced BRD4 protein degradation through the ubiquitin–proteasome system. With dBET1, E3 ligase recognizes, binds, and ubiquitinates BRD4 protein, making it available for subsequent proteasomal degradation. **B** Representative western blot depicts dBET1 (30 mg/kg; i.p. at 4 and 24 h) induces significant BRD4 degradation in the whole hemisphere and cerebral cortex of mice at 48 h after sham surgery. *n* = 4–5 per group, **P < 0.01, ***P < 0.001. **C** Representative western blot shows the BRD4 protein levels in the cerebral cortex of sham and stroke mice at 48 h after surgery. n = 5 per group. ***P < 0.001. **D** Representative western blot depicts dBET1 (30 mg/kg; i.p. at 4 and 24 h after stroke) reduces the protein level of BRD4 in the cerebral cortex under the condition of stroke by tMCAO. ***P < 0.001. Ub, ubiquitin; E1, E2, E3, ubiquitin-activating enzyme E1, E2, E3; BET, bromodomain and extra-terminal domain protein

An ideal approach to block multi-domain protein BRD4 function is to delete BRD4 completely by either targeting protein degradation with proteolysis-targeting chimeras (PROTACs) technique or genetic manipulation [10]. dBET1 is a potent PROTAC molecule, containing the JQ1 moiety for selectively binding BRD4 and a phthalimide moiety for binding the E3 ubiquitin ligase (Fig. 1). Such binding of both moieties allows BRD4 to be tagged with ubiquitin, making it available for subsequent proteasomal degradation. Recent in vitro and in vivo studies have demonstrated that dBET1 can induce potent and selective BRD4 degradation in the mouse brain [18–20]. Considering that the global knockout of BRD4 in mice is lethal, dBET1 investigation is of particular importance in a complete understanding of whether and how BRD4 functions in the brain in heath and disease.

In this study, using a transient focal cerebral ischemia mouse model, we hypothesized that BRD4 degrader dBET1 elicits robust neuroprotective efficacy against stroke damage by mechanisms involving regulating inflammatory and oxidative processes along with preserving BBB integrity. To address this hypothesis, we first validated the ability of dBET1 to degrade BRD4 in vivo. Next, we evaluated the effects of dBET1 on functional and anatomical outcomes after ischemic stroke. We delved into the underlying mechanisms of protection by dBET1 by investigating the effects of BRD4 blockade on ischemia-induced inflammation and oxidative stress. Finally, we studied the effects of post-ischemic treatment with dBET1 on BBB integrity, MMP-9 levels, neutrophil infiltration, cellular adhesion molecule dysregulation, and reactive gliosis in microglia and astrocytes.

## Materials and methods

### Animals

Three-month-old C57BL/6J male mice (Jackson Laboratory) were used for this study. The mice were housed on a 12-h light–dark cycle with ad libitum access to food and water. All animal experiment procedures were performed according to the NIH Guide for the Care and Use of Laboratory Animals and were approved by the University of Florida Institutional Animal Care and Use Committee. The animals were randomly distributed into four experimental groups. All experiments and analysis for this study were conducted in a blinded, randomized, and controlled design to group assignment and treatment. All efforts were made to minimize the animal number and animal suffering. The number of animals for each experiment was calculated based on the power analysis and is stated in each figure or figure legend [21].

### Transient focal cerebral ischemia model

Transient focal cerebral ischemia model was induced by 35-min reversible middle cerebral artery occlusion (tMCAO) with a silicone-coated filament followed by reperfusion, as our group described previously [22, 23]. This transient unilateral cerebral ischemia model generates a reproducible ischemic lesion in the ipsilateral hemisphere. Briefly, each mouse was anesthetized with isoflurane (3% for induction and 1.5–2% for maintenance) in an oxygen/air mixture during surgery. Artificial tear ointment was applied to the eyes for protection and lubrication. The right common carotid artery (CCA), external carotid artery (ECA), and internal carotid artery (ICA) were carefully exposed. The CCA was ligated with a 6–0 silk suture at the proximal portion. A 12-mm length of a 6–0 silicone-coated nylon filament (Doccol, Cat. No. 602123) was gently inserted into the CCA and then advanced into the ICA ~ 9–10 mm from the internal carotid bifurcation until mild resistance was felt and cerebral blood flow (CBF) exhibited a dramatic reduction (> 75% of the baseline value), assessed by a laser Doppler flowmetry (Moor Instruments Ltd). Reperfusion was performed by gently retracting the filament after 35 min of MCA occlusion. The body temperature was maintained at 37 °C by a thermostatically controlled heating pad during the whole surgical procedure. Post-surgery mice were allowed to recover in a temperature- and humidity-controlled chamber. The sham-surgery (sham) mice were subjected to the same surgical procedures except for the MCA occlusion.

### Treatment with BET degrader dBET1

The proteolysis-targeting chimera (PROTAC) dBET1 is a BET protein degrader comprising BET bromodomain antagonist ( +)-JQ1 conjugated to a cereblon E3 ubiquitin ligase ligand [24]. dBET1 (Chemietek, Indianapolis, IN, USA; Cat. #CTDBET1; > 99.5% purity; CAS #1,799,711–21-9) was dissolved in dry dimethyl sulfoxide (75 mg/ml) and stored at − 20 °C as we previous described [19]. The freshly prepared dBET1 working solution or the same volume of vehicle was injected intraperitoneally at 4 and 24 h after the onset of reperfusion. dBET1 was given at a dose of 30 mg/kg. For a 30-g mouse, 12 µl dBET1 stock (or 12 µl dimethyl sulfoxide for vehicle) and 12 µl of 25% Tween 80 in sterile water were mixed and vortexed for 1 min. 476 µl of 11.2% of Captisol® (β-cyclodextrin sulfobutyl ether sodium; Ligand, San Diego, CA, USA) in sterile water was added and thoroughly vortexed again before injection.

### Measurement of infarct volume

The infarct volume was assessed by 2,3,5-triphenyltetrazolium chloride (TTC) staining at 48 h after stroke [25]. Mice were euthanized, transcardially perfused with ice-cold saline, and the brains were harvested. Mouse brains were sliced into six 1-mm-thick coronal sections with a brain matrix. The fourth section, starting from the rostral side, was dissected and immediately frozen in liquid nitrogen and stored in the -80 °C freezer until further molecular biology analyses. The other sections were incubated in a 2% TTC solution (Sigma-Aldrich) at room temperature for 30 min, followed by fixation with 4% paraformaldehyde (PFA, pH 7.4). All rostral sides of the stained sections and the caudal side of the third section (corresponds to the rostral side of the fourth coronal section) were scanned at 600 dpi using an HP Scanjet 8300 scanner (Palo Alto, CA) and saved as a JPEG file. The infarcted area of each section was delineated and determined using Adobe Photoshop CS5, and the overall infarct volume was calculated. The details are described in our previous reports [19, 26].

### Behavioral testing

A battery of behavioral tests, including neurological deficit score, open field locomotor activity, and vertical grid test, were performed to assess the neurological performance of mice at indicated timepoints. Investigators performing the tests and analyses were blinded to group assignment and treatment.

#### Neurological deficit score

The neurological deficits scoring can assess the overall neurological severity and multiple deficits in animal studies of stroke [27, 28]. At 48 h after stroke, mice's neurological deficit score (NDS) was evaluated using six individual tests, including body symmetry, gait, circling behavior, front limb symmetry, compulsory circling, and climbing, as we previously described [29, 30]. Scoring for each test was performed independently by two trained investigators using a 4-point scoring system (0, no deficits; 4, severe deficits). The average score of each mouse from two investigators was used for statistical analysis.

#### Vertical grid test

Vertical grid test is a sensitive method to assess neuromuscular strength and motor coordination in rodents [31, 32]. The vertical grid apparatus is an open frame (55 cm height × 8 cm wide × 5 cm depth) with a wire mesh (0.8 cm × 0.8 cm aperture) on the backside. It is vertically placed in a cage filled with bedding material. Within 1 week prior to and 48 h after tMCAO, each mouse was placed on the top of wire mesh, facing downward, and was allowed to climb down to the cage. The total time to climb down was recorded. If the mouse failed while descending or could not descend down into the cage within 60 s, the performance was expressed as the maximum duration of 60 s. Each mouse performed 3 trials with at least 5-s intervals.

#### Open field test

The open field test is used to assess the locomotor activity of mice. Within 1 week prior to and at 48 h after tMCAO, the spontaneous locomotor activity of mice was measured in an open field paradigm with an automated video tracking system (Anymaze software, Stoelting, Wood Dale, IL) as we described previously [19]. Mice were individually placed in an open field chamber (40 × 40 × 40 cm) with grey sidewalls and allowed to explore for 10 min. The total traveled distance was used as the indices of motor/exploratory behavior of each mouse. The open field arena was cleaned with 70% ethanol between tests.

### Immunohistochemistry and immunofluorescence

Each mouse was anesthetized and transcardially perfused with saline followed by 4% paraformaldehyde (pH 7.4) in PBS. The brain was removed, post-fixed, and cryoprotected in 30% sucrose (w/v) and sliced into 30-μm-thick coronal sections on a semi-automatic vibrating microtome (Compresstome® VF310-0Z; Precisionary Instruments, Natick, MA). Immunohistochemistry and immunofluorescence stainings were performed using standard protocols [33, 34]. The primary antibodies were rabbit polyclonal ionized calcium-binding adapter protein 1 (Iba1; 1:5000; catalog #019–19,741, Wako Bioproducts, Richmond, VA), rabbit polyclonal glial fibrillary acidic protein (GFAP; 1:3000; catalog #Z0334, DAKO, Carpinteria, CA), horse anti-mouse IgG (biotinylated; 1:200; catalog #BA-2000, Vector Laboratories, Burlingame, CA), purified rat anti-mouse Ly-6G (1:200; catalog #127,602, Biolegend, San Diego, CA), and goat anti-mouse intercellular adhesion molecule-1 (ICAM-1/CD54; 1:1000; catalog #AF796, R&D Systems, Minneapolis, MN). Sections were washed and incubated with Elite ABC-HRP Kit for Ly-6G and IgG stainings (catalog #PK-6100, Vector Laboratories, Burlingame, CA) or appropriate secondary antibodies for other stainings on the following day. The following secondary antibodies were used: goat anti-rabbit (1:2000; catalog #5450–0010, SeraCare, Gaithersburg, MD) and donkey anti-goat Alexa Fluor Plus-594 (1:500; catalog #A32758, Life Technologies, Grand Island, NY). For immunohistochemistry, the immunoreaction was visualized using a 3,3-diaminobenzidine chromogen solution (DAB substrate kit; Vector Laboratories). The brightfield images were captured by ScanScope CS and analyzed using ImageScope software (Aperio Technologies, Vista, CA) or ImageJ software. Immunofluorescence images were captured by an automated Axio Scan.Z1 slide scanner and analyzed using Zen 2.3 software (Zeiss, Berlin, Germany) or ImageJ software. The immunostaining signals were quantified in a 100 × 100 µm square applied at indicated brain regions in the figures. Three consecutive coronal sections were quantitatively analyzed and provided an average value. We counted Iba1-positive cells area (%), GFAP-positive astrocytes area (%), IgG immunointensity, Ly-6G positive cells per field (i.e., 200 µm × 200 µm square), and ICAM-1 immunointensity in indicated brain regions.

### Protein extraction and western blot

Protein extraction and western blot were performed as previously described [25]. The deeply anesthetized mice were transcardially perfused with ice-cold saline. Harvested brain tissues (peri-infarct/contralateral cortex or hemispheres) were collected and stored in the − 80 °C freezer. Tissues were homogenized in lysis buffer, and total protein concentration was determined using the PierceTM BCA assay kit (catalog #23,227, Thermo Scientific, Rockford, IL). Samples were aliquoted and stored at − 80 °C freezer until further analysis. An equal amount of protein was separated using 4–20% polyacrylamide gradient gels (BioRad, Hercules, CA) and transferred to nitrocellulose membranes. After blocking with 5% skim milk, the membranes were incubated overnight with rabbit anti-BRD4 (1:2000; Catalog #A301-985A50; Bethyl Laboratories, Montgomery, TX, USA), rabbit anti-zona occludens 1 (ZO-1; 1:1000; catalog #61–7300, Life Technologies, Grand Island, NY), rabbit anti-Occludin (1:1000; catalog #ab167161, Abcam, Cambridge, MA), rabbit anti-MMP-9 (1:500; catalog #sc-6841-R, Santa Cruz Biotechnology, Dallas, Texas), mouse-4-hydroxynonenal (4-HNE; 1:2000, catalog #MAB3249, R&N systems, Minneapolis, MN), mouse anti-gp91^phox^ (1: 500; catalog #611,414, BD Biosciences, San Jose, California), rabbit anti-glutathione peroxidase 1 (GPX1; 1:1000; catalog #ab22604, Abcam, Cambridge, MA), rabbit anti-Superoxide dismutase 2 (SOD2; 1:2,000, catalog #ab13534, Abcam, Cambridge, MA), and mouse anti-β-actin (1:10,000; catalog #A1978, Sigma-Aldrich, St. Louis, MO). The membranes were then washed with TBST three times at 5 min intervals, incubated with goat anti-rabbit IRDye 800CW (1:30,000; Li-Cor, Lincoln, NE) or donkey anti-mouse IRDye 680LT (1:40,000; Li-Cor) secondary antibodies for 1 h at room temperature. Membranes were scanned with an Odyssey infrared scanning system (Li-Cor), and quantification analyses of immunoreactive bands were performed using ImageJ software. Unedited immunoblots are provided in Additional file 1: Figure S1. For gp91^phox^, we optimized the immunoblotting conditions and found a better signal when gels are run under non-reducing conditions (Additional file 2: Figure S2).

### RNA isolation and quantitative real-time PCR

Mice were euthanized and transcardially perfused with ice-cold saline, and the brains were collected and sliced into six 1-mm-thick coronal sections with a brain matrix. The cortical tissue was dissected from the fourth section and was stored at − 80 °C until RNA extraction. Total RNA from the cortical tissue was isolated using a modified method of acid guanidinium thiocyanate–phenol–chloroform extraction [19, 35]. RNA concentration and purity were determined by a Take3 Micro-Volume Plate Reader (Biotek Instruments, Winooski, VT). Quantitative real-time PCR was performed in a total reaction volume of 10 μL using Luna® Universal One-Step RT-qPCR Kit (catalog # E3005, New England BioLabs, Ipswich, MA) according to the manufacturer’s protocol. Reactions were run in a BioRad CFX96 Touch Real-Time PCR Detection System. The primer sequences were included in Table 1. Each reaction was performed in triplicate, and the relative expression value for each target gene was calculated using the ΔΔCt method after normalization to the housekeeping gene *Ywhaz*.

**Table 1:** Mouse primer sequences for real-time PCR analysis

Gene	Primer sequence (5′ to 3′)		Accession number
	Forward	Reverse	
*Ccl2*	CATCCACGTGTTGGCTCA	AACTACAGCTTCTTTGGGACA	NM_011333
*Cxcl1*	CCAAACCGAAGTCATAGCCA	GTGCCATCAGAGCAGTCT	NM_008176
*Cxcl10*	ATTTTCTGCCTCATCCTGCT	TGATTTCAAGCTTCCCTATGGC	NM_021274
*Il-1β*	GACCTGTTCTTTGAAGTTGACG	CTCTTGTTGATGTGCTGCTG	NM_008361
*Mmp9*	GACATAGACGGCATCCAGTATC	GTGGGAGGTATAGTGGGACA	NM_013599
*Tnf-α*	AGACCCTCACACTCAGATCA	TCTTTGAGATCCATGCCGTTG	NM_013693
*Ywhaz*	TGTTCTAGCCTGTTTCCCCG	ACGATGACGTCAAACGCTTC	NM_011740
*Il-6*	AGCCAGAGTCCTTCAGAGA	TCCTTAGCCACTCCTTCTGT	NM_031168

Gene abbreviations: Ccl2, chemokine (C–C motif) ligand 2, Cxc1–C–X–C motif chemokine ligand; Cxcl10, C–X–C motif chemokine 10; Il-1β, interleukin-1β; Mmp9, matrix metalloproteinase-9; Tnfα, tumor necrosis factor α; Ywhaz, Tyrosine 3-Monooxygenase/Tryptophan 5-Monooxygenase Activation Protein Zeta; Il-6, Interleukin 6

### Statistical analysis

PRISM software (GraphPad V.6) was used to perform the statistical analyses. D’Agostino–Pearson normality test was utilized to confirm Gaussian distribution before performing parametric statistical tests. An independent unpaired Student’s *t* test was performed for comparison of two groups. Multiple comparisons were made using one-way or two-way ANOVA followed by Bonferroni’s post hoc test. All data were expressed as group mean ± SEM, and *P* ≤ 0.05 was considered statistically significant. The number of samples for each experiment is stated in each figure or figure legend. All studies were analyzed with investigators blinded to treatments and groups.

## Results

### dBET1 administration induces potent BRD4 degradation in the mouse brain

Our initial objective was to confirm whether BRD4 PROTAC molecule dBET1, at dose of 30 mg/kg (i.p. at 4 and 24 h after sham-surgery), crosses the blood–brain barrier and induces effective BRD4 degradation in the mouse brain. Our preliminary data (Additional file 3: Figure S3) revealed that, at dose of 30 mg/kg but not 10 mg/kg, dBET1 induced prominent degradation of BRD4 in the cerebral cortex of naïve mice. Therefore, 30 mg/kg was used in current study. Following 48 h after sham-surgery, we found that dBET1 treatment led to a statistically significant reduction of BRD4 protein in either cerebral cortex or the whole hemisphere of adult C57BL/6 mice, measured by western blot (Fig. 1B). As shown in Fig. 1C, stroke induced by tMCAO led to a dramatic reduction in BRD4 protein expression level at 48 h after surgery. In addition, dBET1 treated mice also exhibited lower protein level of BRD4 under stroke condition (Fig. 1D). These findings further confirmed robust BRD4 degradation efficacy by dBET1 in the mouse brain under either normal or stroke conditions, in agreement with the BRD4 PROTAC dBET1 mechanism-induced BRD4 protein degradation through the ubiquitin–proteasome system (Fig. 1A).

### dBET1 ameliorates neurological deficits induced by ischemic stroke

**Fig. 2:**
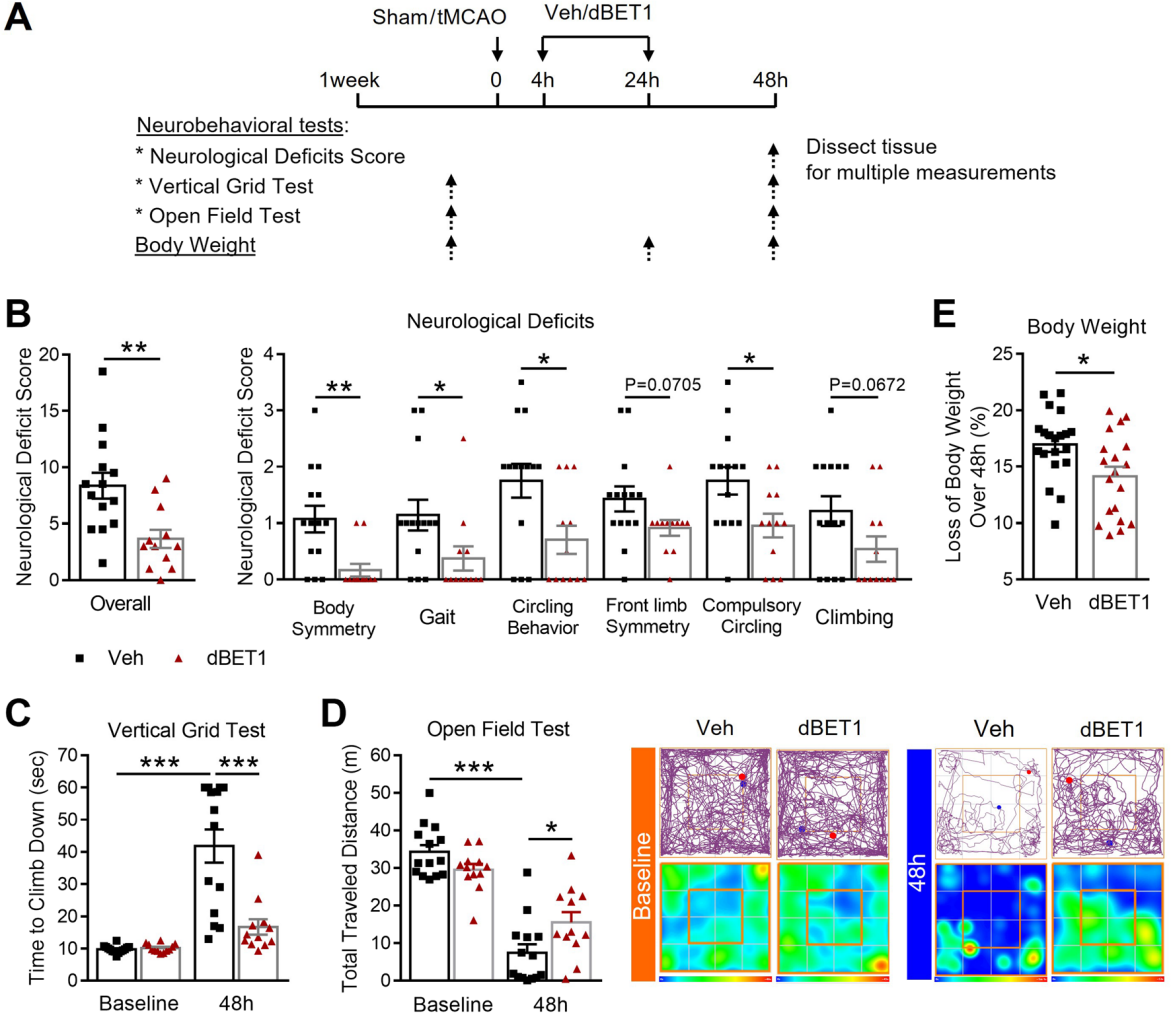
dBET1 ameliorates ischemia-induced neurological deficits. **A** Schematic illustration of the experimental design. Adult male C57BL/6 mice were injected intraperitoneally with dBET1 (30 mg/kg) at 4 and 24 h after 35-min transient middle cerebral artery occlusion (tMCAO); the body weight and neurobehavioral tests were examined at indicated timepoints. **B** Neurological deficit score was assessed using six individual functional tests, where a lower value indicates a better function. The dBET1 treated ischemic mice exhibited a dramatically reduced neurological deficit score compared to controls in overall or individual scores. Veh: *n* = 14; dBET1: *n* = 12. **C** No significant baseline difference was detected between groups in the vertical grid test. The dBET1 treated mice used much less time (about 54% reduction) to climb down from the top of the apparatus than vehicle controls, which is closer to baseline level. Veh: *n* = 14; dBET1: *n* = 12. **D** No significant baseline difference was found between groups in the total traveled distance in the open field test. After stroke, the mice traveled much less distance, which is more evident in the dBET1 treated group. Representative tracking maps and corresponding heat maps indicate the time of animal spent per location in the open field paradigm. Veh: *n* = 14; dBET1: *n* = 12. **E** dBET1 significantly decreased the loss of body weight over 48 h after tMCAO. Veh: *n* = 14; dBET1: *n* = 12. *P < 0.05, **P < 0.01, ***P < 0.001. Veh, vehicle

Our previous study have demonstrated the neuroprotective effect of dBET1 in a permanent ischemic stroke mouse model [19]. To investigate dBET1 effects on stroke outcomes in a transient focal ischemia mouse model by 35 min-tMCAO, multiple neurobehavioral and histological outcomes were evaluated at 48 h (Fig. 2). As shown in Fig. 2B, dBET1 significantly alleviated ischemia-induced severe neurological deficits compared to vehicle controls, indicated by neurological deficit score (NDS). Such improvements were observed in all six individual assessments of NDS, including body symmetry, gait, climbing, circling behavior, front limb symmetry, and compulsory circling. Following tMCAO, mice from both groups underwent a rapid functional deterioration in locomotor activity and neuromuscular strength and motor coordination, indicated by the total traveled distance in the open field test and the time to climb down in the vertical grid test, respectively (Fig. 2C, D). Strikingly, dBET1 ameliorated ischemia-induced functional deterioration. No obvious difference at baseline behavioral performance in the above measures was observed between groups. In addition, dBET1 treatment dramatically reduced the body weight loss over 48 h after stroke (Fig. 2E). Taken together, the results of the behavioral assays demonstrate the protective effect of dBET1 on ischemia-induced acute neurological deficits after tMCAO.

### dBET1 reduces acute ischemic brain damage

**Fig. 3:**
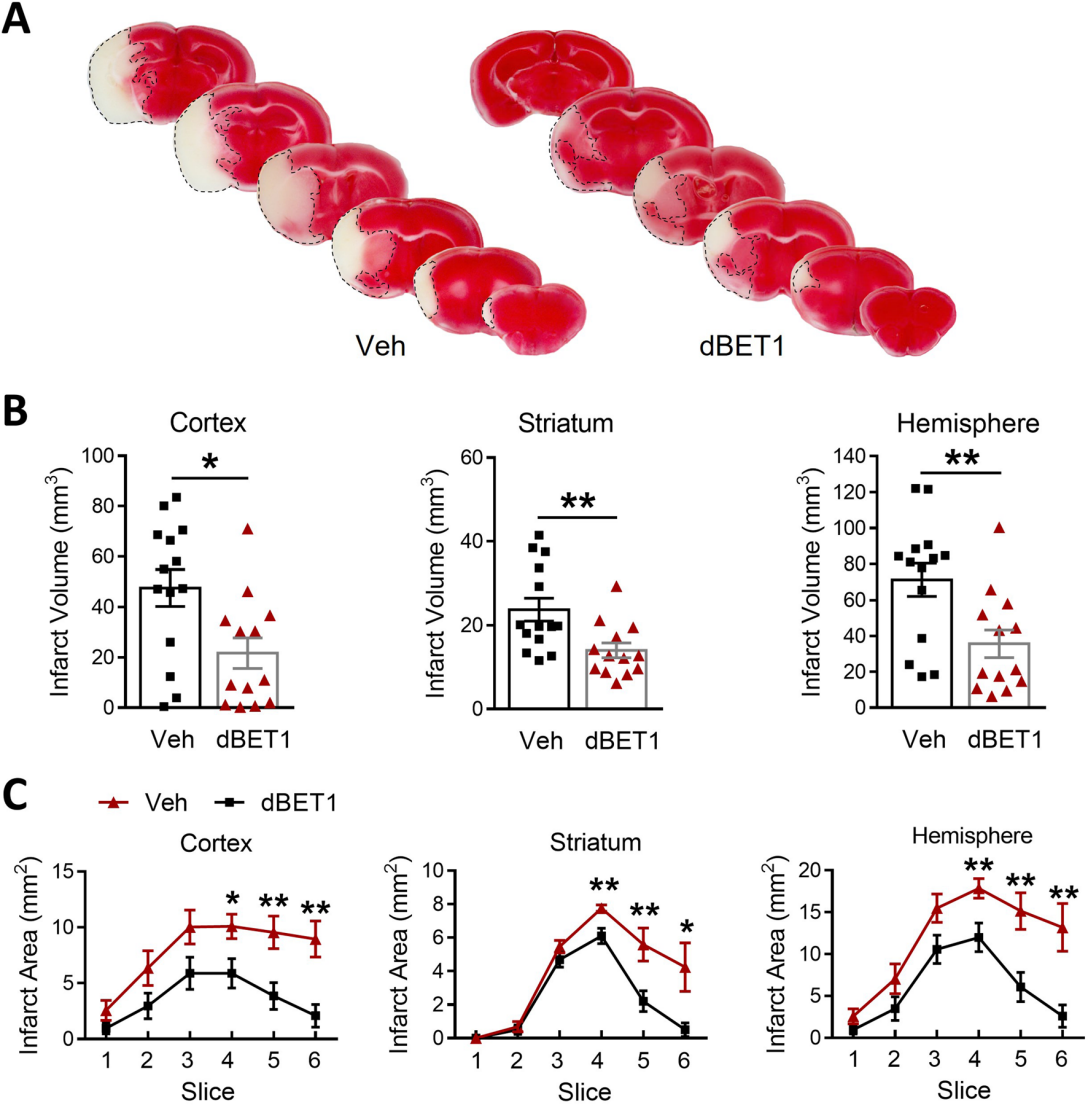
dBET1 reduces infarct volume after stroke. **A** Representative images of TTC stained 1-mm thick coronal brain slices of vehicle- and dBET1-treated mice at 48 h after tMCAO. Quantitative analyses of the overall infarct volume (**B**) or detailed infarct area of each brain slice (**C**) were performed. The cortex, striatum, and hemisphere infarct volumes **B** were significantly reduced in the dBET1 group than the vehicle-treated control group. Analysis of infarct area per slice shows that the decreases in infarct volume are seen throughout the hemisphere. Veh: *n* = 14; dBET1: *n* = 13. *P < 0.05, **P < 0.01. Veh, vehicle; TTC, 2,3,5-triphenyl-2H-tetrazolium chloride

Functional deficits caused by stroke are often associated with the extent of histological damage in affected brain regions. Consistent with the behavioral performances above, dBET1-treated mice exhibited smaller infarct volume than vehicle-treated mice 48 h after tMCAO (cortex: 47.5 ± 7.3% vs 21.7 ± 6.1%, *P* < 0.05; striatum: 23.7 ± 2.7% vs 14.0 ± 1.8%, *P* < 0.01; hemisphere: 71.3 ± 9.2% vs 35.7 ± 7.8%, *P* < 0.01; Fig. 3), as measured by TTC staining. The reduction in infarct volume was detected in all observed brain regions. Further examinations of 1-mm slices revealed a reduction in infarct size from brain regions observed in slices 4–6 from the dBET1-treated group. These results are indicative of dBET1 protection against ischemic brain lesion.

### dBET1 reduces pro-inflammatory mediators and enhances the antioxidant capacity after stroke

**Fig. 4:**
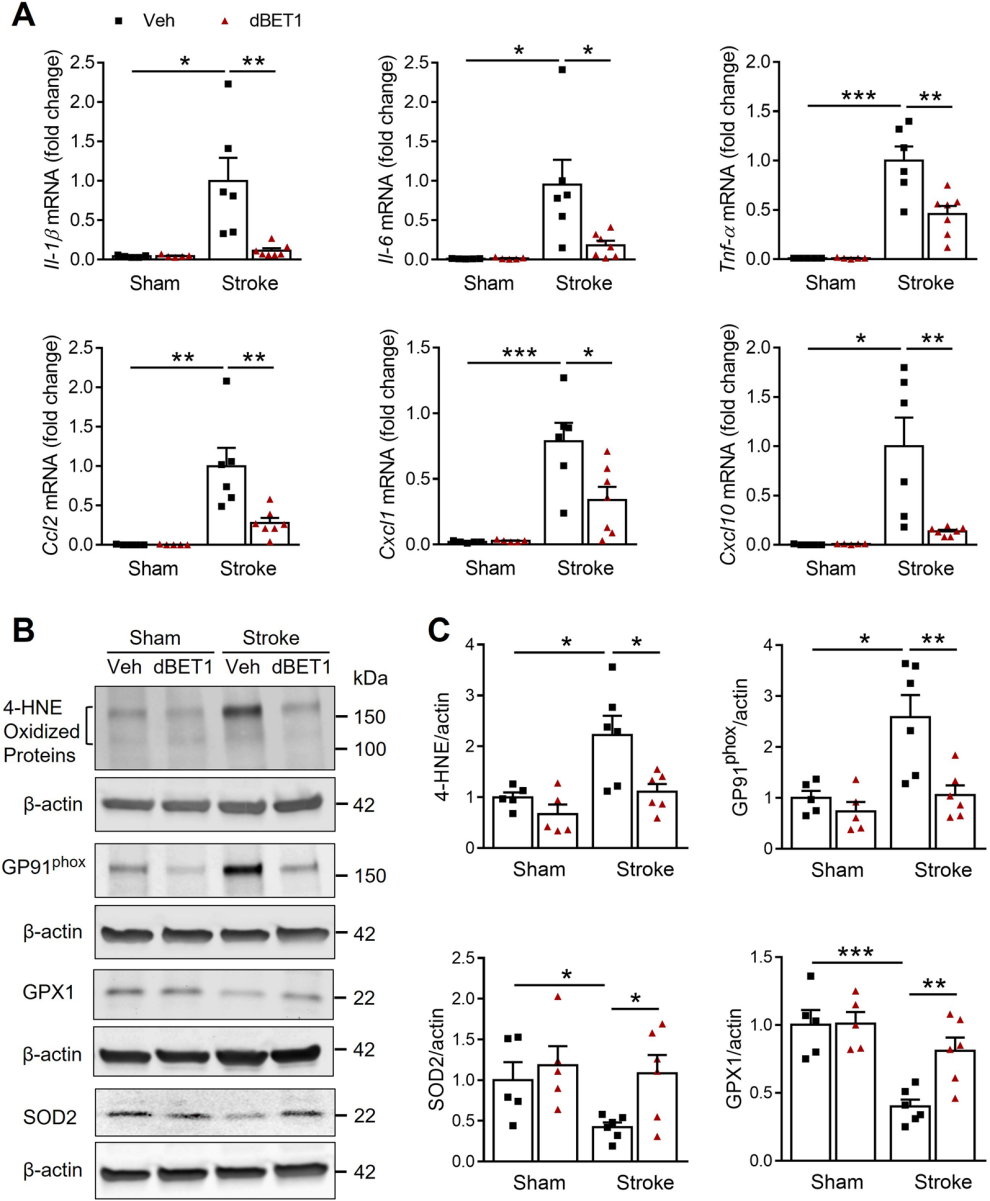
dBET1 protects against inflammatory and oxidative damage in the ischemic cortex. **A** Effect of dBET1 on neuroinflammation following tMCAO was examined by measuring the mRNA levels of pro-inflammatory cytokines and chemokines IL-1β, IL-6, TNF-α, Ccl2, Cxcl1, and Cxcl10 in peri-infarct regions of cortex at 48 h after tMCAO. Compared to sham controls, stroke evoked a prominent increase in the above markers, which were significantly suppressed by dBET1 treatment. *n* = 5 per sham group, *n* = 6 stroke veh group, *n* = 7 stroke dBET1 group. *P < 0.05, **P < 0.01, ***P < 0.001. **B** Representative western immunoblots showed the expression levels of 4-HNE-modified proteins and NADPH oxidase isoform NOX2 (GP91^phox^), two oxidative stress markers, and SOD2 and GPX1, two antioxidant proteins, in the ischemic cortex at 48 h after tMCAO and sham controls. *β*-actin was used as a loading control. **C** Quantitative analysis in **B** showed that stroke led to a significant increase in 4 HNE and GP91^phox^ and a decline in SOD2 and GPX1, which were reduced by dBET1. *n* = 5 per sham group, *n* = 6 per stroke group. *P < 0.05, **P < 0.01, ***P < 0.001

In an initial effort to identify the underlying mechanism of dBET1 protection, we hypothesized that dBET1 could reduce inflammation and oxidative stress in the ischemic cortex at 48 h after tMCAO (Fig. 4). Real-time quantitative PCR measurements suggested the prominent elevation of multiple pro-inflammatory cytokines and chemokines, including IL-1β, IL-6, TNF-α, CCL2, CXCL1, and CXCL10 after stroke. Post-ischemic dBET1 treatment significantly reduced the upregulation of these markers. Western blot analysis revealed that the stroke insult led to a marked increase of oxidative damage levels, indicated by 4-hydroxy-2-nonenal (4-HNE)-modified proteins and GP91^phox^-containing NADPH oxidase (NOX2), and decrease of antioxidative enzyme levels, indicated by superoxide dismutase 2 (SOD2) and glutathione peroxidase 1 (GPX1). In contrast, dBET1 significantly reduced 4-HNE and GP91^phox^ levels and enhanced SOD2 and GPX1 levels. In addition, no significant difference at baseline in any markers above was observed between sham groups. Together, these findings suggest that reducing inflammation and oxidative damage may contribute to dBET1 protection in stroke.

### dBET1 protects against ischemia-induced BBB integrity disruption, MMP-9 activity increase, and neutrophil infiltration

**Fig. 5:**
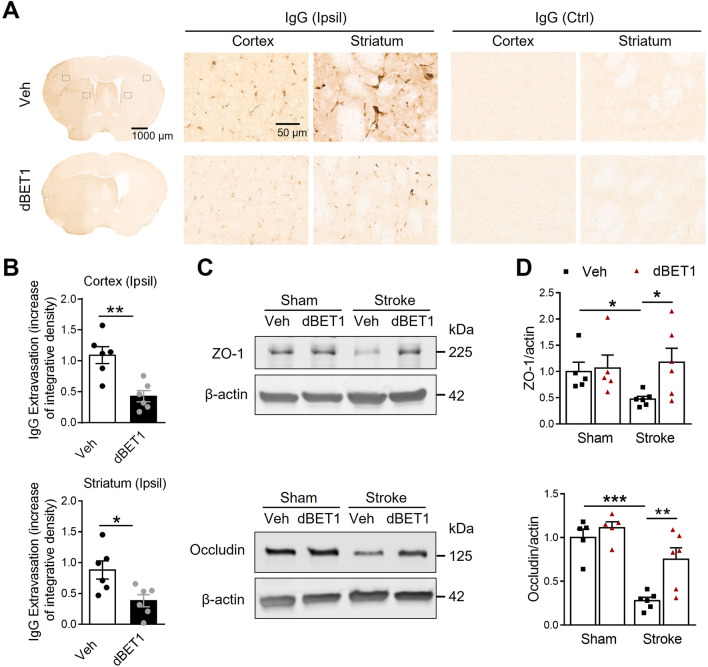
dBET1 reduces ischemia-induced blood–brain barrier breakdown. **A** Representative images of IgG stain in the post-stroke mouse brain sections, showing the leakage of serum immunoglobulin G (IgG) through the disrupted blood–brain barrier (BBB) at 48 h after tMCAO; and open squares in the left image indicate the peri-infarct areas of the ipsilateral (Ipsil) cortex and striatum, as well as the contralateral (Ctrl) side, used for micrographic examination. No IgG stain signal is observed in the contralateral side of mouse brains. Apparent IgG stains are widely detected in the peri-infarct area, which is dramatically reduced in the dBET1 group. **B** Quantifications of IgG signals in **A** showed that dBET1 significantly reduced the IgG extravasation compared to veh controls. *n* = 6 per group. *P < 0.05, **P < 0.01. **C** Representative immunoblots for tight junction proteins ZO-1 and occludin, two important structural components of the BBB, in homogenates from the ischemic cortex and sham controls. β-actin was used as a loading control. **D** Quantitative analysis shows that stroke resulted in marked degradation of ZO-1 and occludin in the ischemic cortex than sham controls. In contrast, such degradation of these two proteins was significantly attenuated in the dBET1 stroke group. *n* = 5 per sham group, *n* = 6 per stroke group. *P < 0.05, **P < 0.01, ***P < 0.001

Inflammatory and oxidative damage exacerbates ischemia-induced BBB breakdown [2]. Therefore, we explored whether improving stroke outcomes by dBET1 was associated with the preservation of BBB integrity. At 48 h after tMCAO, a dramatic increase in BBB permeability, indicated by immunoglobulin G (IgG) extravasation, was observed in the ipsilateral cerebral cortex and striatum compared with the contralateral side of ischemic animals, while dBET1 treatment significantly reduced such damage (Fig. 5). Tight junction proteins in endothelial cells are critical structural components of the BBB. Post-ischemic treatment with dBET1 prevented ischemia-induced degradation of tight junction proteins, indicated by the preservation of zona occludens (ZO)-1 and occludin protein levels (Fig. 5C, D).

**Fig. 6:**
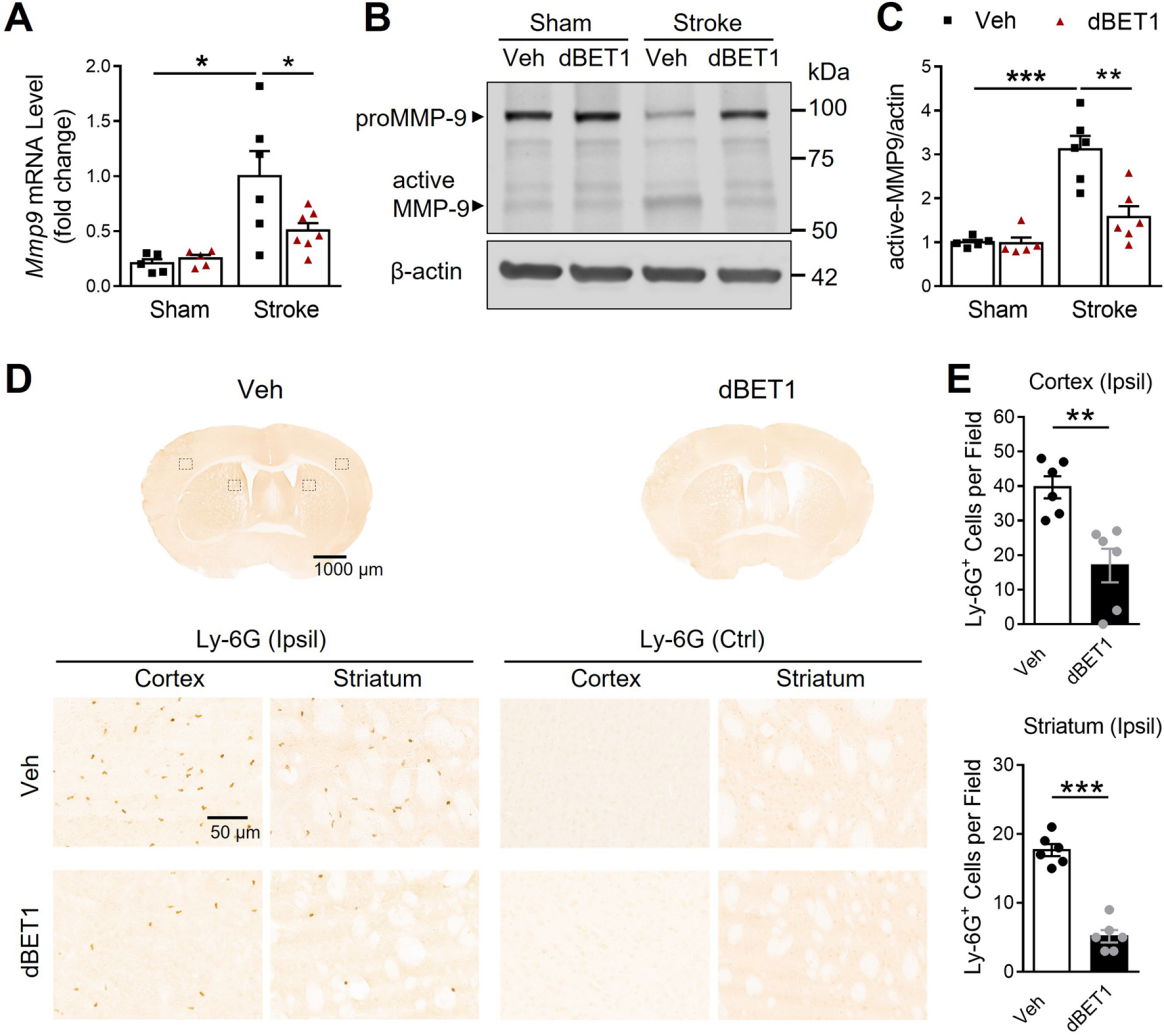
dBET1 attenuates ischemia-induced MMP-9 increase and neutrophil infiltration. **A** Matrix metalloproteinase-9 (MMP-9) mRNA level of the ischemic and sham cortex was measured by real-time PCR. Stroke led to a dramatic increase in MMP-9 mRNA level, which was significantly reduced by dBET1. *n* = 5 per sham group, *n* = 6 stroke veh group, *n* = 7 stroke dBET1 group. *P < 0.05. **B** Representative immunoblot for MMP-9 level in homogenates from the ischemic cortex and sham controls. **C** dBET1 significantly reduced active MMP-9 protein level in the ipsilateral (Ipsil) cerebral cortex compared to the veh group. *n* = 5 per sham group, *n* = 6 per stroke group. **P < 0.01, ***P < 0.001. **D** Representative images of staining for Ly-6G, a specific marker of neutrophils, in the post-stroke mouse brain sections at 48 h after tMCAO, showing the neutrophilic infiltration; and open squares in the left image indicate the peri-infarct areas of the ipsilateral cortex and striatum, as well as the contralateral (Ctrl) side, used for micrographic examination. No Ly-6G stain signal is detected in the contralateral side of both groups. Accumulated Ly-6G staining is widely distributed in the peri-infarct area, which is much lower in the dBET1 group. per field, 200 µm × 200 µm square. **E** Quantifications of Ly-6G in **D** showed that the dBET1 group exhibits reduced expression level compared to veh group (> 50% in ischemic cortex and > 70% in ischemic striatum. *n* = 6 per group. **P < 0.01, ***P < 0.001

Activated MMP-9 following stroke is known to degrade tight junction proteins and contribute to BBB disruption [36]. Compared to sham controls, stroke resulted in a sharp increase in MMP-9 mRNA and protein levels in the ischemic cortex, which was significantly reduced by dBET1 (Fig. 6). No significant baseline difference was found between sham groups. Infiltrating neutrophils, the primary source of active MMP-9 in the ischemic brain, also contribute to BBB damage [37]. Invasion of neutrophils into the ischemic brain was quantified by counting the number of cells positive for Ly-6G, a highly specific neutrophil marker, at 48 h after tMCAO. We observed a dramatic infiltration of neutrophils into the ischemic side of cortical and striatal regions compared to the non-ischemic side, which was significantly attenuated in the dBET1 group. Together, these findings reveal that dBET1 profoundly preserves the BBB integrity and protects against MMP-9 activation and neutrophil infiltration after stroke.

### dBET1 reduces ICAM-1 levels after stroke

**Fig. 7:**
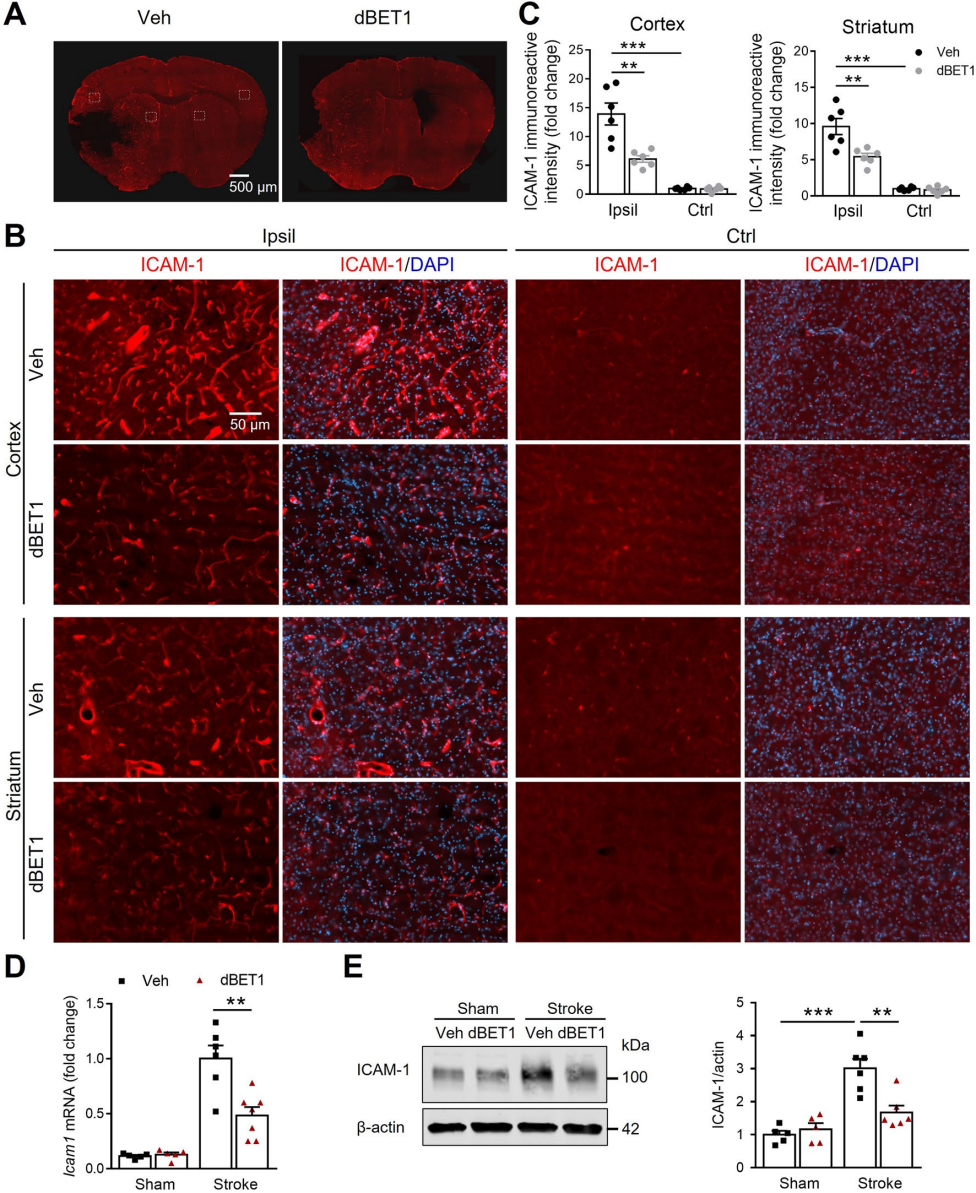
dBET1 attenuates ischemia-induced dysregulation of cellular adhesion molecule ICAM-1. **A** Representative images of endothelial intercellular adhesion molecule 1 (ICAM-1) of the post-stroke mouse brains at 48 h after tMCAO; and open squares indicate the ipsilateral (Ipsil) peri-infarct cortex and striatum areas and corresponding contralateral (Ctrl) areas for micrographic examination (**B**). **C** Quantifications of ICAM-1 signals intensity in **B**. dBET1 reduced ischemia-induced prominent increase of ICAM-1 expression level in either cortex or striatum of the ischemic hemisphere. *n* = 6 per group. **P < 0.01, ***P < 0.001. **D** Representative real-time PCR for ICAM-1 mRNA level in the ischemic cortex and sham controls. *n* = 5 per sham group, *n* = 6 veh-stroke group, *n* = 8 dBET1-stroke group. **P < 0.01. **E** Representative western blots for ICAM-1 level in the ischemic cortex and sham controls. dBET1 significantly attenuated ischemia-induced ICAM-1 upregulation in mRAN and protein levels compared to the corresponding veh controls. *n* = 5 per sham group, *n* = 6 per stroke group. **P < 0.01, ***P < 0.001.

Intercellular adhesion molecule 1 (ICAM-1) is known to exacerbate BBB permeability during stroke and play a significant role in the cascade of immune cell transmigration into the brain parenchyma [38, 39]. We next determined whether dBET1 can modulate the expression of ICAM-1, a critical mediator of peripheral immune cell infiltration, in the ischemic brain at 48 h after tMCAO (Fig. 7). Analysis of ICAM-1 immunoreactive intensity showed a nearly 10–13-fold increase in ICAM-1 levels in the ischemic cortex and striatum compared to the corresponding non-ischemic side in vehicle-treated animals. Notably, dBET1 significantly attenuated the sharp upregulation of ICAM-1 induced by stroke. Real-time quantitative PCR and Western blot analyses showed that stroke led to a marked upregulation of ICAM-1 mRNA and protein levels, which were significantly attenuated by dBET1 treatment. In addition, basal expression levels of ICAM-1 did not reveal significant differences. Together, these results suggest that the beneficial effects of dBET1 in stroke are associated, in part, with reduced ICAM-1 levels.

### dBET1 attenuates ischemia-induced reactive gliosis

**Fig. 8:**
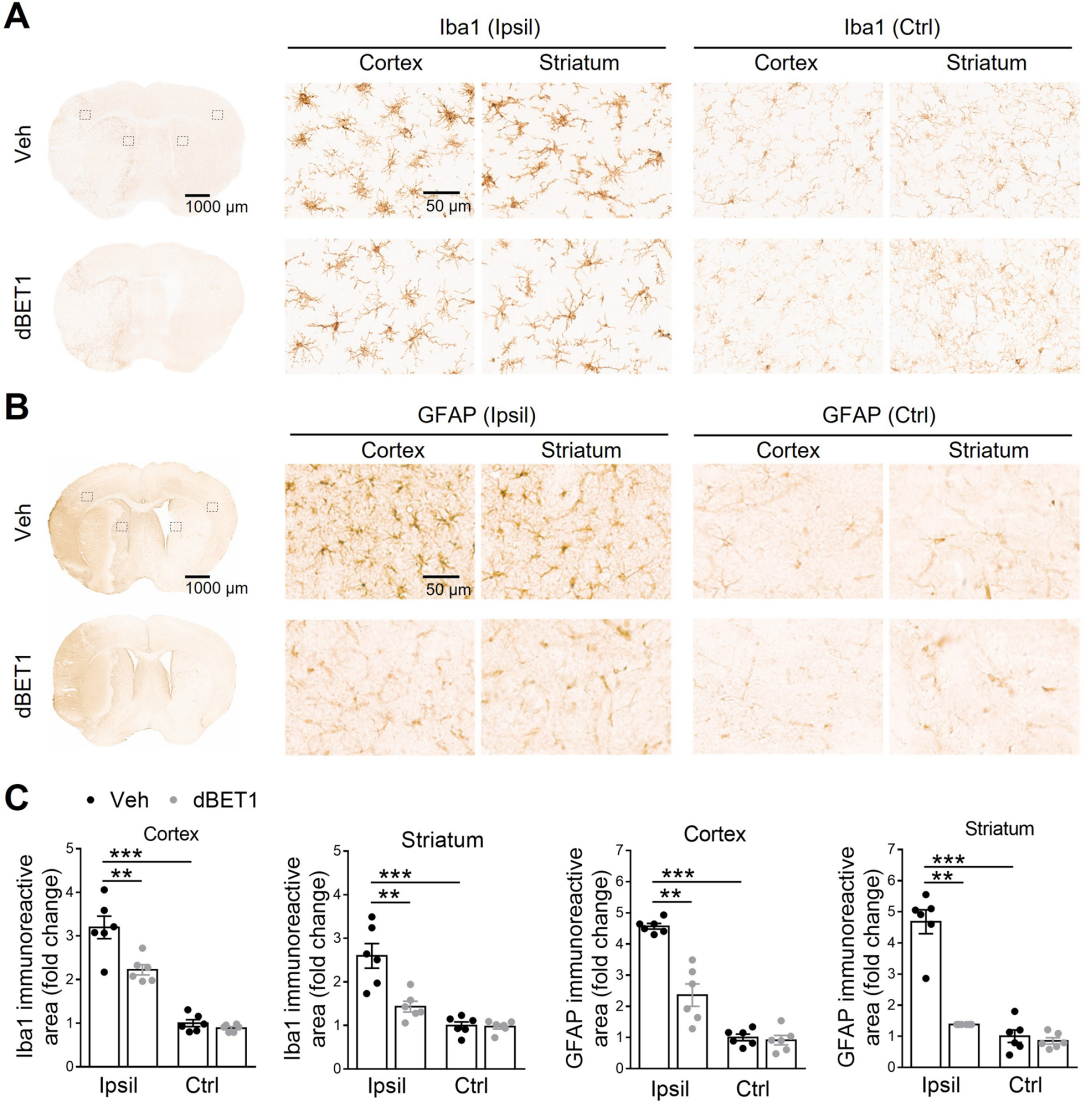
dBET1 attenuates ischemia-induced reactive gliosis. Representative images of Iba1 positive microglia (**A**) and GFAP positive astrocyte (**B**) in the cortex and striatum of mice at 48 h after tMCAO; and open squares in the left image indicate the peri-infarct areas of the ipsilateral (Ipsil) cortex and striatum, as well as the contralateral (Ctrl) side, used for micrographic examination. **C** Quantifications of the areas of Iba1 and GFAP signals in **A **and **B** respectively. Acute stroke evoked reactive gliosis, the response of glial cells to ischemic insults, in the peri-infarct area, in microglia (characterized by hypertrophic soma with thickened and retracted processes) and astrocytes (characterized by hypertrophic somas and highly stained processes). dBET1 treatment remarkably attenuated the deteriorative progression of reactive gliosis in microglia and astrocytes compared to veh controls. In addition, no obvious difference was detected in both markers in the contralateral cortex and striatum regions. *n* = 6 per group. *P < 0.05, **P < 0.01. Veh, vehicle

Glial cells are a major source of inflammatory and oxidative mediators. Following stroke, microglia and astrocytes undergo remarkable changes in gene expression, morphology, and proliferation, termed reactive gliosis [40, 41]. Finally, we examined the effect of dBET1 on reactive gliosis in the peri-infarct areas of the cortex 48 h after tMCAO by immunostainings with the microglial marker Iba1 and the astrocyte marker GFAP (Fig. 8). In both sham groups of mice, microglia covered the cortex and striatum regions in a regular distribution pattern, most of which remain in a static state with small somas and fine processes. In contrast, stroke evoked the activation and proliferation of microglia in both groups, as featured by hypertrophic soma with thickened and retracted processes. Notably, the extent of this reactive microgliosis was significantly attenuated in dBET1-treated ischemic mice than in corresponding controls given the vehicle. Similar to our observation of microglia morphology, no significant difference in reactive astrogliosis was observed between sham groups. In contrast, stroke injury evoked significant activation of astrocytes in both groups, as characterized by hypertrophic somas and highly stained processes, which was remarkably attenuated in the dBET1 group. More degenerated astrocytes with broken down somas were observed in vehicle-treated ischemic mice, indicating that the severe deteriorative progression was dramatically delayed by dBET1. It should be pointed out that Iba1 also labels monocyte-derived macrophages following stroke. Together, these findings suggest that dBET1 delays stroke-triggered reactive gliosis progression in microglia and astrocytes, which may impact tissue preservation and functional outcomes.

## Discussion

**Fig. 9:**
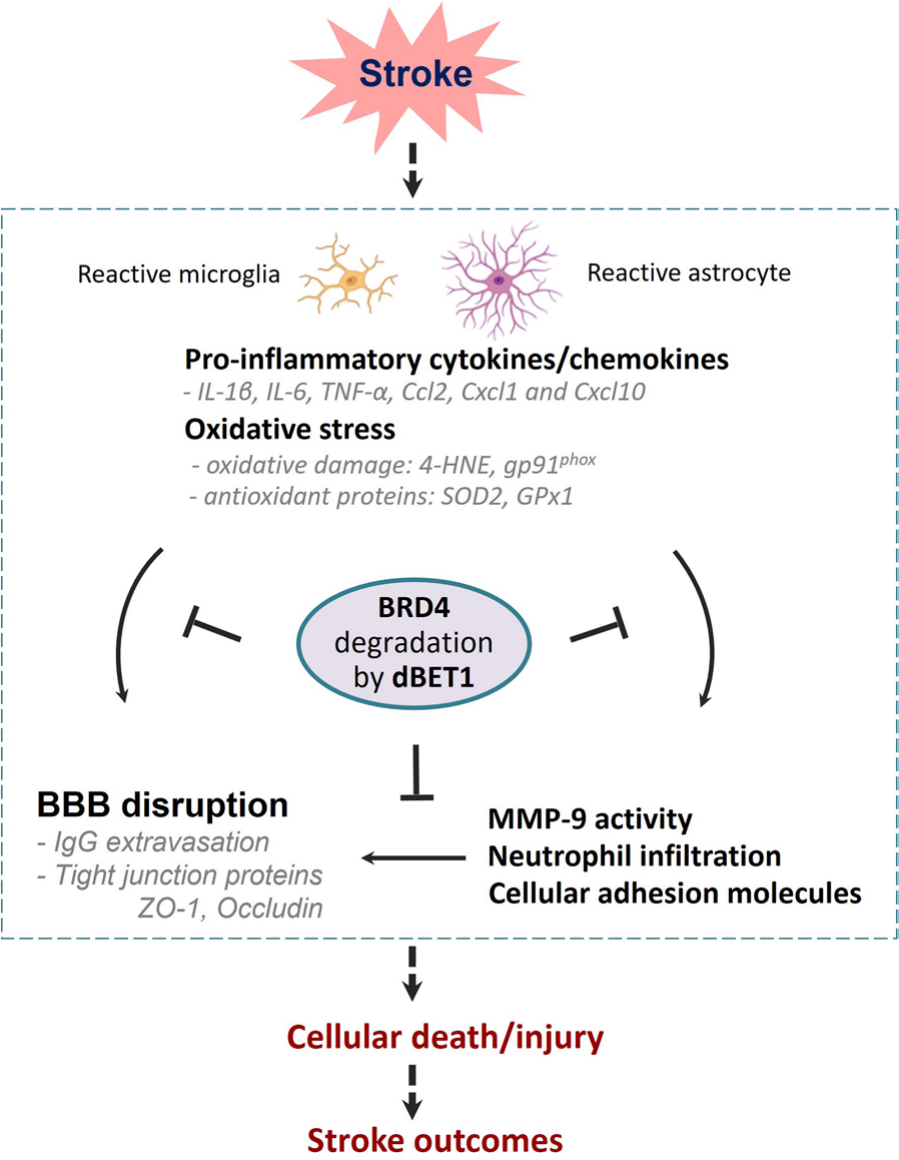
Schematic summarizing the molecular mechanisms underlying the protection of dBET1 in ischemic stroke

This study provides several lines of evidence supporting dBET1 neuroprotection against ischemia-induced neurological deficits and brain damage through regulating inflammation and oxidative stress as well as preserving BBB integrity. dBET1 administration induced effective BRD4 degradation in the brain. dBET1 remarkably ameliorated ischemia-induced severe neurological deficits and brain lesion volume (56.2 ± 7.9% reduction). Meanwhile, we found that dBET1 reduced inflammation and oxidative stress in the ischemic cortex, indicated by multiple pro-inflammatory mediators (IL-1β, IL-6, TNF-α, CCL2, CXCL1, and CXCL10) and oxidative and antioxidative markers (4-HNE, gp91^phox^, SOD2, and GPx1). Stroke triggered BBB breakdown and MMP-9 activation, neutrophil infiltration, and increased expression of ICAM-1, which were profoundly attenuated by dBET1. The deteriorative progression of reactive gliosis in microglia and astrocytes was delayed by dBET1. A summarized schematic of the effects of dBET1 on transient ischemic stroke is presented in Fig. 9.

### BRD4 degradation by dBET1 elicits robust neuroprotective efficacy against stroke outcomes

To date, recognition for the roles of BRD4 in neurological conditions is limited [5]. Because PROTAC molecule dBET1 contains a BET bromodomain binding moiety JQ1 with much higher selectivity and affinity for BRD4 than other BET members, BRD4 functional regulation is the primary effect of dBET1 [5, 42–44]. PROTAC technique is a leading strategy for targeting protein degradation through the ubiquitin proteosome system. Considering that the global loss of BRD4 is lethal in mice, dBET1 studies help identify in vivo BRD4 protein function and its contribution to stroke. Previously, we observed that dBET1 could significantly improve functional and histological outcomes in a permanent ischemic stroke mouse model [19]. Here, we demonstrated that, in a transient ischemic stroke mouse model, dBET1-treated animals exhibited ameliorated neurological deficits and a reduction of brain lesions. Notably, the protection conferred by dBET1 is seen when the first dose is given 4 h after the onset of reperfusion, which is a clinically achievable therapeutic window for intervention. Our data further advances our understanding of the protective role of BRD4 degradation by dBET1 in ischemic stroke.

### BRD4 protein degradation by dBET1 is associated with reduced neuroinflammation and oxidative stress, preservation of BBB integrity, and reduced reactive gliosis

Inflammation and oxidative stress are crucial stroke pathological mechanisms, which critically determine the extent of brain injury, functional impairment, and prognosis. BRD4 shows promising potential in direct and indirect regulation of these two factors, emerging as a therapeutic target for stroke and other neurological disorders. Following stroke, oxidative stress reactions result in hyperactivation of inflammatory responses and further exacerbate stroke injury [45]. We found that dBET1 administration markedly reduced the levels of multiple pro-inflammatory cytokines and chemokines IL-1β, IL-6, TNF-α, CCL2, CXCL1, and CXCL10. Substantial evidence shows that several pro-inflammatory cytokines, such as IL-1β, IL-6, and TNF-α, as well as other potential cytotoxic molecules, are released in response to ischemic insults [46]. Previous reports showed that JQ1-treated animals exhibit a marked reduction in the expression of pro-inflammatory mediators IL-1β, IL-6, IL-17, IL-18, and TNF-α in the brain in rodent ischemic stroke models [5, 47]. Emerging evidence suggests that BRD4 may act as a critical transcriptional regulator of NF-κB-dependent inflammatory gene expression, such as CCL2, CXCL1, and CXCL10 [5]. The interaction between BRD4 protein and the NF-κB pathway will be explored in our near future study, which will further elucidate BRD4-dependent molecular mechanisms contributing to neurovascular damage in stroke. Ischemic insults trigger the rapid overproduction of reactive oxygen species (ROS) and other free radicals that overwhelm the endogenous antioxidant capacity, including antioxidant enzymes, superoxide dismutase, and the antioxidant glutathione, eventually causing damage to lipids, proteins, and DNA [48]. After stroke, dBET1 dramatically reduced oxidative damage levels revealed by decreased 4-HNE, a lipid peroxidation product, and gp91^phox^, a major source of oxygen radical generation, and improved the expression levels of antioxidant enzymes SOD2 and GPx1. These two enzymes are required for eliminating free radicals and reducing by-products of lipid peroxidation reactions [30, 49]. The effect of dBET1 on oxidative stress could be associated with Nrf2 signaling and its target genes. BET proteins were recently reported as Nrf2 signaling repressors, which involves Keap1-dependent regulation and Keap1-independent regulation [5, 13, 50]. Additional studies are necessary to further our understanding of the complex biology of BET proteins and their crosstalk with the Nrf2 pathway.

BBB highly restricts transcellular movement of solutes and paracellular diffusion across the barrier either into or out of the brain, which plays essential roles in cerebral homeostasis. BBB disruption following secondary injuries such as inflammation and oxidative stress are known to exacerbate ischemia-induced brain injury and limit functional recovery [16, 51]. Brain endothelial cells comprising the BBB interact with pericytes, astrocytes, neurons, microglia, and perivascular macrophages in the neurovascular unit. The excessive accumulated inflammatory and oxidative mediators following stroke cause endothelial dysfunction, which leads to BBB breakdown, indicated by increased BBB permeability and the degradation of BBB structural components, including the endothelial tight junction proteins ZO-1 and occludin. dBET1 treatment attenuated the exacerbated BBB permeability, indicated by the extravasation of IgG, the degradation of tight junction proteins ZO-1 and occludin. Post-ischemic dBET1 treatment significantly reduced MMP-9 levels, neutrophil infiltration into the ischemic brain, and dysregulated levels of cellular adhesion molecules (e.g., ICAM-1) after stroke. Inflammatory and oxidative stimuli induce recruitment and activation of inflammatory cells, including neutrophils, increased MMPs (mainly MMP-9 activity), and increased ICAM-1. This cascade of events lead to endothelial dysfunction and BBB damage [25, 52]. Infiltrating neutrophils are a primary source of active MMP-9 that causes degradation of tight junction proteins in the ischemic brain [25, 46, 52]. Decreased MMP-9 activation by dBET1 could reduce the infiltration of active MMP-9-laden neutrophils. Inflammatory and oxidative damage also promote adhesion molecule expression on cerebral endothelial cells and mediate leukocyte recruitment, an essential component of the inflammatory response in the ischemic brain. ICAM-1 is upregulated on the surface of the endothelial cells and promotes the adhesion and extravasation of leukocytes.

Microglia and astrocytes are the main cell types in the regulation of ischemia-induced neuroinflammation and oxidative stress, which respond most rapidly to brain insults by morphologically changing and proliferating and through the enhanced release of various inflammatory mediators. They act through reactive gliosis processes and display cellular hypertrophy, proliferation, and migration in response to ischemic insults including inflammatory and oxidative damage [53]. Following ischemic insults, reactive astrocytes assist in buffering changes in extracellular ion homeostasis, such as glutamate, altering the osmoregulation, counteracting the development of brain edema, and repairing the integrity of the BBB [41]. Studies suggest that Nrf2 downstream target genes that encode for phase II defense enzymes and antioxidant proteins, such as heme oxygenase-1 (HO1) and NAD(P)H dehydrogenase [quinone] 1 (NQO1), are particularly enriched in glia cells, supporting the key role of reactive gliosis process in regulating inflammatory and oxidative responses following ischemia [16, 30]. dBET1 attenuated the damaging progression of reactive gliosis in microglia and astrocytes in the peri-infarct areas, which conferred protection against ischemic insult. Following stroke, glial cells are activated, and circulating immune cells invade the ischemic brain; both resident and infiltrating cells orchestrate the post-stroke inflammatory response. Recent reports revealed that the increased density of microglia in response to brain injury correlates with both the proliferation of endogenous resident microglia and the active recruitment of microglia progenitors from the blood [54, 55]. Ischemic stroke causes local inflammation, which involves the activated resident microglia and infiltrating immune cells, such as neutrophils and monocyte-derived macrophages [56, 57]. Both cell types above can be labelled by Iba1 antibody, suggesting that dBET1 might play a key role in the immunity regulation in stroke.

As limitations of the current study, it should be pointed out that we only observed dBET1 effects on acute stroke outcomes. The longer observation timepoints could help to demonstrate the efficacy of dBET1 on stroke and further elucidate the underlying neuroprotection mechanisms of BRD4 degradation by dBET1. In this study, we demonstrated that the protective mechanisms of dBET1 involve regulation of inflammation and oxidative stress and maintenance of BBB integrity. For our near future experiments, we will explore whether dBET1 can regulate nuclear factor-κB (NF-κB)-dependent inflammatory gene expression and Nrf2-downstream target antioxidant genes, two critical independent regulatory mechanisms in the context of stroke [5].

In conclusion, this is the first study to demonstrate that BRD4 degradation by dBET1 ameliorates neurological deficits and brain injury by mechanisms involving regulating inflammation and oxidative stress and preserving BBB integrity in the context of transient ischemic stroke. Another novel aspect of the present study is the finding that a delayed administration of dBET1 significantly impacts stroke outcomes. These findings strongly suggest that targeting BRD4 is a promising therapeutic strategy to ameliorate ischemic brain injury and improve functional outcomes following ischemic stroke.

## Supplementary Information

**Additional file 1:** **Figure S1.** Unedited Western blots.

**Additional file 2: Figure S2.** Optimized immunoblotting conditions for detecting gp91phox signal. Immunoblot for gp91phox-containing NADPH oxidase under nonreduced and reduced conditions in the cortex of adult mouse brain subjected to stroke. Results show better signal for gp91phox (~ 170 kDa) under the non-reducing conditions. CXI, cortex ipsilateral to stroke; CXC, cortex contralateral to stroke.

**Additional file 3: Figure S3.** Dose–response effects of dBET1 on the degradation of BRD4 in the mouse cerebral cortex. Quantitative analyses of immunoblot data for BRD4 protein in the cortex of mice at 6 h after the intraperitoneal injection of vehicle or dBET1 (10, 30 mg/kg). **P < 0.01. n.s., Not Significant.

## Data Availability

The original data that support the findings of this study are available from the corresponding author upon reasonable request.
