# Supplementary figures and images for: Targeted BRD4 protein degradation by dBET1 ameliorates acute ischemic brain injury and improves functional outcomes associated with reduced neuroinflammation and oxidative stress and preservation of blood–brain barrier integrity
Source: J Neuroinflammation. 2022 Jun 27;19:168. doi: 10.1186/s12974-022-02533-8 (PMC9237998; doi:10.1186/s12974-022-02533-8)

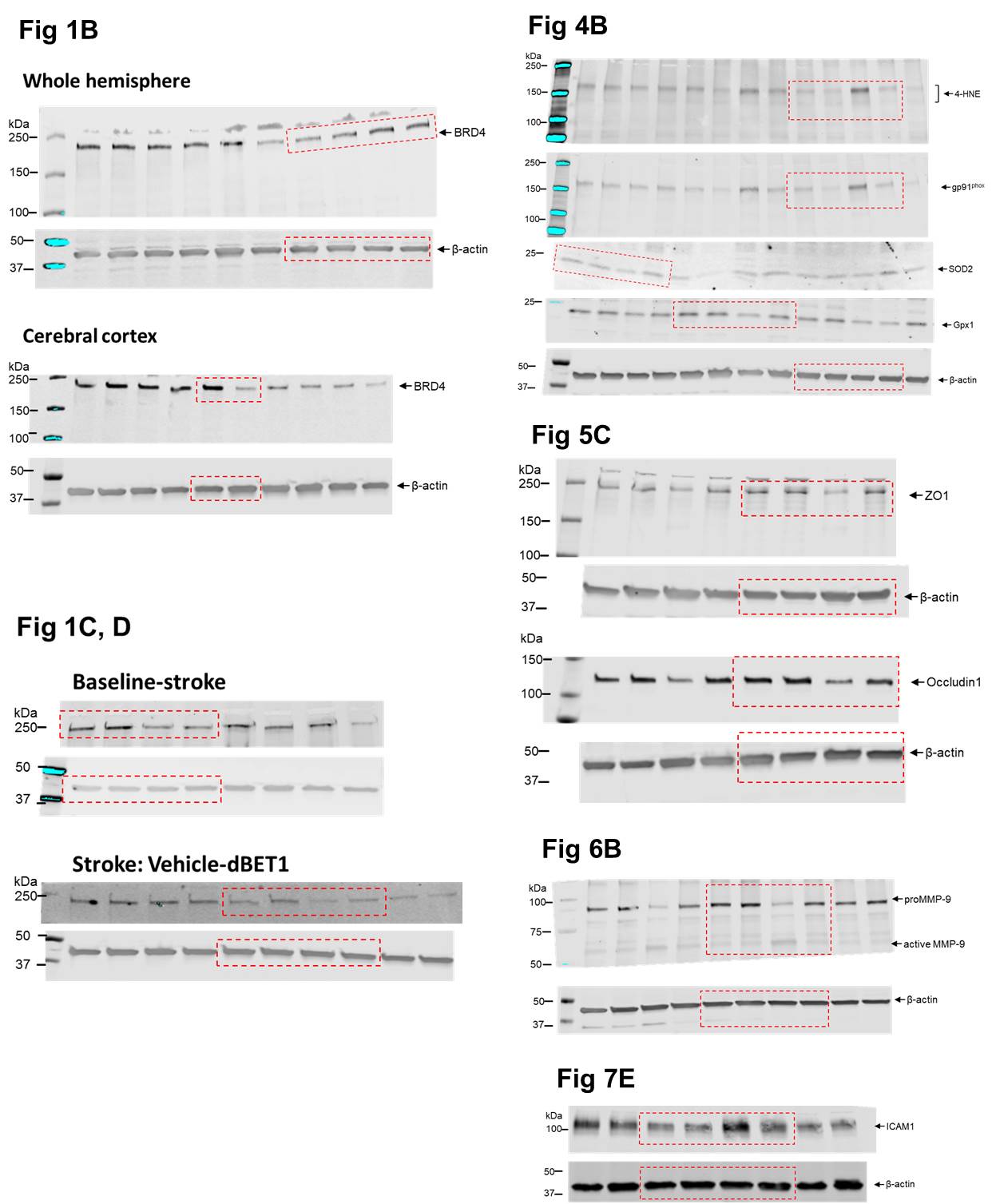

Supplement: Supplementary file 1 — Additional file 1: Figure S1. Unedited Western blots. [file 12974_2022_2533_MOESM1_ESM.jpg]

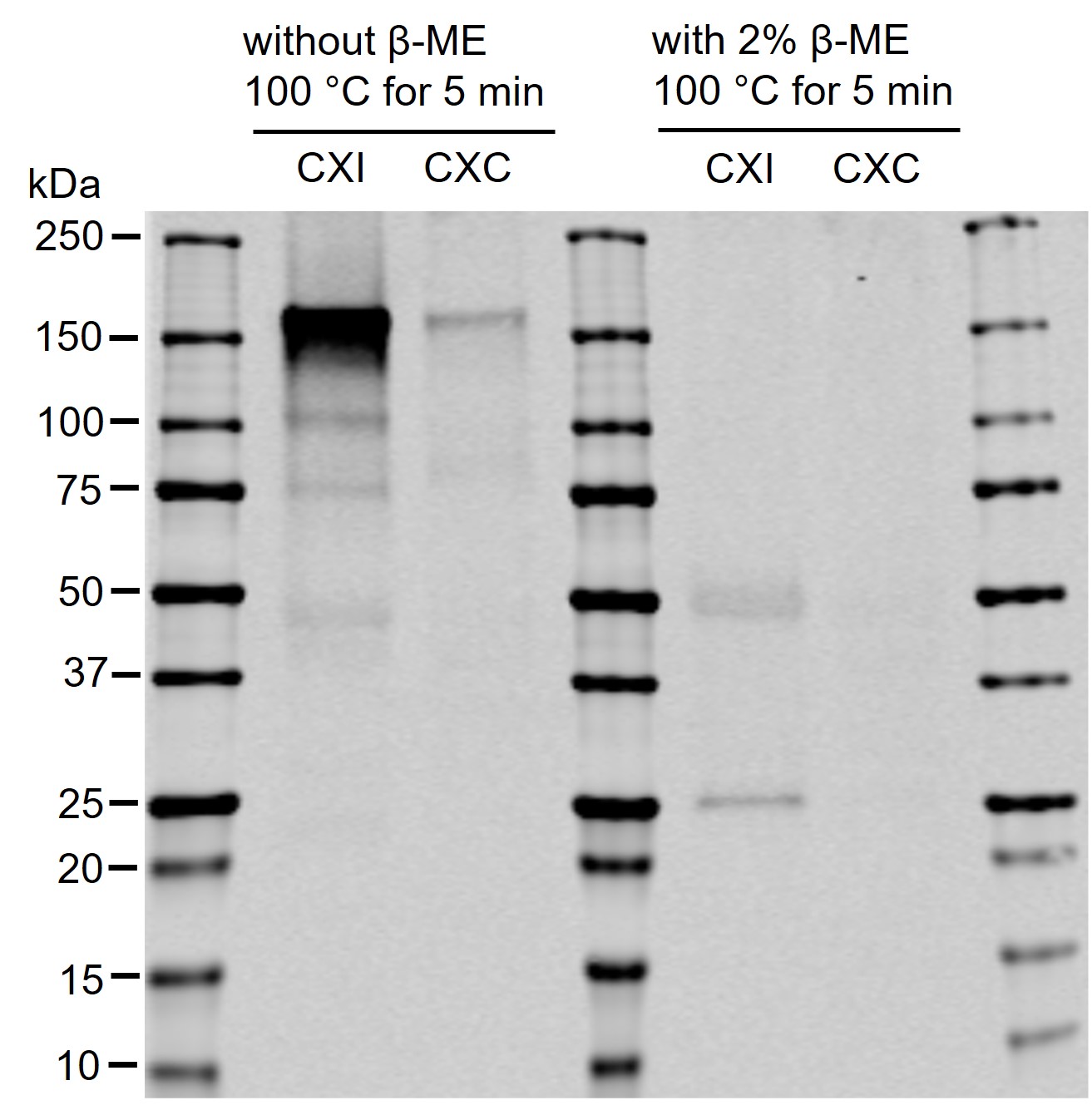

Supplement: Supplementary file 2 — Additional file 2: Figure S2. Optimized immunoblotting conditions for detecting gp91phox signal. Immunoblot for gp91phox-containing NADPH oxidase under nonreduced and reduced conditions in the cortex of adult mouse brain subjected to stroke. Results show better signal for gp91phox (~ 170 kDa) under the non-reducing conditions. CXI, cortex ipsilateral to stroke; CXC, cortex contralateral to stroke. [file 12974_2022_2533_MOESM2_ESM.jpg]

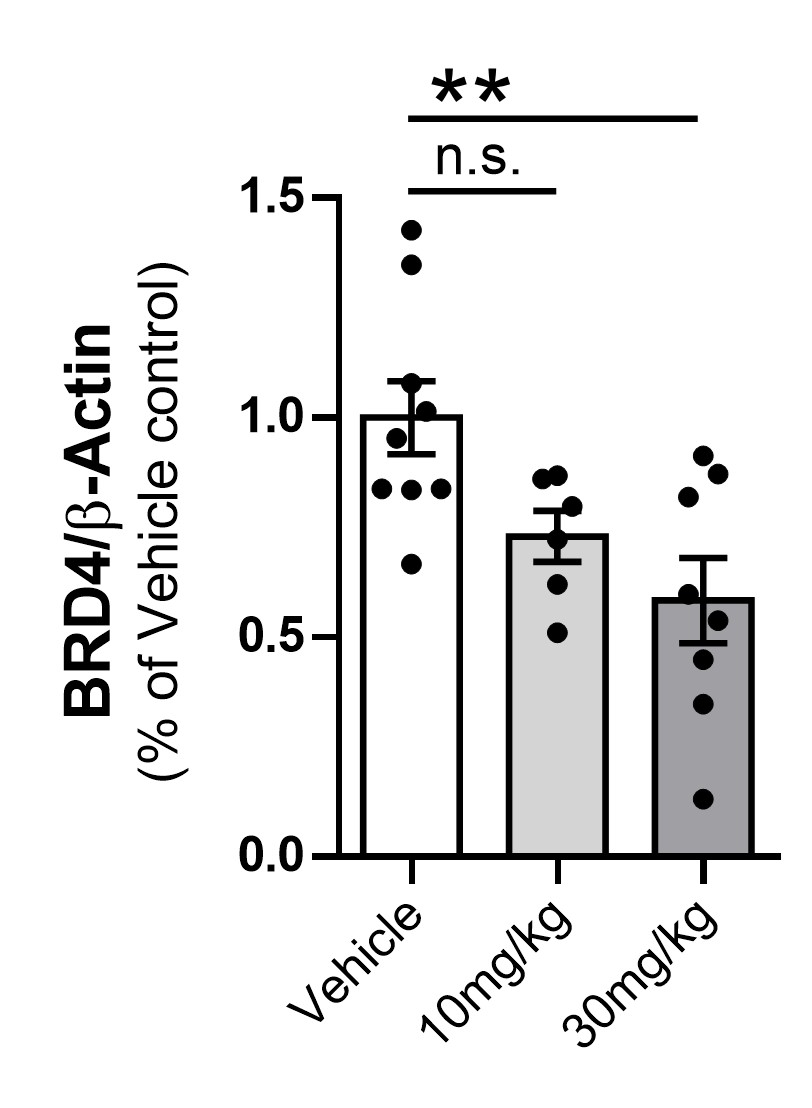

Supplement: Supplementary file 3 — Additional file 3: Figure S3. Dose–response effects of dBET1 on the degradation of BRD4 in the mouse cerebral cortex. Quantitative analyses of immunoblot data for BRD4 protein in the cortex of mice at 6 h after the intraperitoneal injection of vehicle or dBET1 (10, 30 mg/kg). **P < 0.01. n.s., Not Significant. [file 12974_2022_2533_MOESM3_ESM.jpg]
